# DNA-vaccination via tattooing induces stronger humoral and cellular immune responses than intramuscular delivery supported by molecular adjuvants

**DOI:** 10.1186/1479-0556-6-4

**Published:** 2008-02-07

**Authors:** Dana Pokorna, Ivonne Rubio, Martin Müller

**Affiliations:** 1 Department of Experimental Virology, Institute of Hematology and Blood Transfusion, Prague, Czech Republic; 2 Deutsches Krebsforschungszentrum, Heidelberg, Germany

## Abstract

Tattooing is one of a number of DNA delivery methods which results in an efficient expression of an introduced gene in the epidermal and dermal layers of the skin. The tattoo procedure causes many minor mechanical injuries followed by hemorrhage, necrosis, inflammation and regeneration of the skin and thus non-specifically stimulates the immune system. DNA vaccines delivered by tattooing have been shown to induce higher specific humoral and cellular immune responses than intramuscularly injected DNA. In this study, we focused on the comparison of DNA immunization protocols using different routes of administrations of DNA (intradermal tattoo versus intramuscular injection) and molecular adjuvants (cardiotoxin pre-treatment or GM-CSF DNA co-delivery). For this comparison we used the major capsid protein L1 of human papillomavirus type 16 as a model antigen. L1-specific immune responses were detected after three and four immunizations with 50 μg plasmid DNA. Cardiotoxin pretreatment or GM-CSF DNA co-delivery substantially enhanced the efficacy of DNA vaccine delivered intramuscularly by needle injection but had virtually no effect on the intradermal tattoo vaccination. The promoting effect of both adjuvants was more pronounced after three rather than four immunizations. However, three DNA tattoo immunizations without any adjuvant induced significantly higher L1-specific humoral immune responses than three or even four intramuscular DNA injections supported by molecular adjuvants. Tattooing also elicited significantly higher L1-specific cellular immune responses than intramuscularly delivered DNA in combination with adjuvants. In addition, the lymphocytes of mice treated with the tattoo device proliferated more strongly after mitogen stimulation suggesting the presence of inflammatory responses after tattooing. The tattoo delivery of DNA is a cost-effective method that may be used in laboratory conditions when more rapid and more robust immune responses are required.

## Introduction

DNA vaccination has experienced great progress since the initial discovery of the spontaneous transfection of myocytes after intramuscular delivery of plasmid DNA in saline solution in 1990 [1]. Yet, intramuscular administration by simple injection of DNA is considered to be one of the less effective routes of DNA vaccination. The transfection of cells after single syringe injection of naked DNA is a rather inefficient process and various improvements using different physical, biochemical and biological methods have been made. Among the commonly used methods of DNA vaccination, the highest efficacy was achieved after *in vivo *electroporation and gene gun delivery [2].

Tattooing is an invasive procedure involving a solid vibrating needle that repeatedly punctures the skin, wounding both the epidermis and the upper dermis in the process and causing cutaneous inflammation followed by healing [3]. Modified tattooing devices have been used in medical research for the delivery of various materials to the skin for different purposes, e.g. bleomycin for the treatment of hypertrophic scars [4], viruses to induce papillomas in mice and rabbits [5], pigments to study processes associated with cosmetic tattooing [3] and DNA for prospective gene therapy of skin disorders or vaccination [6-8]. Techniques based on multiple puncturing (up to 15 punctures) are used in human medicine to assess immune responses [9,10] as well as for vaccination [11,12]. As tattooing involves a much larger area of the skin than intradermal injection, it offers an advantage of potentially transfecting more cells [13]. Gene expression after DNA tattooing has been shown to be higher than that after intradermal injection [7,8] and gene gun delivery [8]. DNA vaccines delivered by tattoo were able to induce both cellular [6,7] and humoral antigen-specific responses [6,8]. Compared to intra-muscular injection of DNA, delivery of DNA by tattooing seems to produce different gene expression patterns. In one study, tattooing of 20 μg DNA resulted in at least ten times lower peak values of gene expression than intramuscular injection of 100 μg DNA. Gene expression after tattoo application peaked after six hours and vanished over the next four days, while the intramuscular injection of DNA resulted in high levels of gene expression peaking after one week and remaining detectable up to one month [6]. Despite lower dose of DNA and decreased gene expression, DNA delivered by tattoo induced higher antigen-specific cellular as well as humoral immune responses than intramuscular DNA injection [6,8].

In this work, we evaluated the effect of two adjuvants, cardiotoxin and plasmid DNA carrying the gene for the mouse granulocyte-macrophage colony-stimulating factor (GM-CSF), on the efficiency of a DNA vaccine delivered either by tattoo or intramuscular needle injection. As a model antigen, we used a codon modified gene encoding the L1 major capsid protein of the human papillomavirus type 16 (HPV16) that has been shown to be highly immunogenic in our previous experiments using intramuscular administration of DNA in combination with cardiotoxin pre-treatment [14]. Our results indicate that molecular adjuvants substantially enhance the efficiency of the HPV16 L1 DNA vaccine when administered intramuscularly. However, the delivery of the HPV16 L1 DNA in the absence of adjuvants using a tattoo device elicited much stronger and more rapid humoral and cellular immune responses than intramuscular needle delivery together with molecular adjuvants.

## Methods

### Animals

Eight-week-old female C57BL/6 (H2^b^) mice were purchased from Charles River (Sulzfeld, Germany) and kept under specific pathogen-free conditions at the animal facilities of the German Cancer Research Center in compliance with the regulations of the Germany Animal Protection Law.

### Plasmids

Plasmid pUF3L1h [14] carrying the humanized HPV16 L1 gene under the control of the human cytomegalovirus immediate-early promoter (pCMV) was used for the induction of antigen-specific immune responses in the DNA immunization experiments. The L1 protein expression of pUF3L1h has been shown to be substantially increased due to the codon optimization.

The plasmid pBSC/GM-CSF (kindly provided by M. Smahel, Institute of Hematology and Blood Transfusion, Prague, the Czech Republic) was used as an adjuvant in the DNA immunization experiment. This plasmid contains the sequence coding for the mouse GM-CSF that was excised from the plasmid pBK-GM [15] by *Xho*I and *Sal*I restriction enzymes and ligated into the *Xho*I-site of the plasmid pBSC [16]. The production of GM-CSF was confirmed by transfecting 293T cells with the pBSC/GM-CSF plasmid and analyzing lysates using the mouse GM-CSF ELISA kit (OptEIA™, BD Biosciences Pharmingen, San Diego, CA, USA). The adjuvant effect of pBSC/GM-CSF plasmid has been evaluated in our previous immunization experiments [17].

### DNA immunization

Plasmid DNA was purified from *E. coli *DH5α using CsCl equilibrium density centrifugation and dissolved in TE buffer to a final concentration of 5 mg/ml. Anesthetized mice were immunized with DNA four times, on days 0, 14, 28 and 98. Each mouse received 50 μg of plasmid pUF3L1h (6 groups) or pBSC/GM-CSF (control group) in one immunization dose. Two groups of mice received a mixture of 50 μg pUF3L1h DNA and 50 μg pBSC/GM-CSF DNA per animal in a single dose. For intramuscular delivery, the DNA was injected into the tibia anterior muscle of the right leg in a final volume of 50 μl PBS. Tattooed DNA was delivered in 10 μl TE buffer for single plasmid administration or 20 μl TE buffer for the mixture of plasmids in one or two drops to the shaved skin at the dorsum followed by tattoo with a 7-linear tattoo needle using a commercial tattoo machine (Rotary 12000 PL, Bortech Tattoogrosshandel, Wuppertal, Germany). The tattoo device was adjusted to allow exposure of only 1–2 mm of the needle tip beyond the barrel guide. The depth of 1–2 mm for tattooing of the mouse skin was shown to result in the immediate location of tattooed inks mainly in the dermis and to a lower extent in the epidermis [3]. A skin surface area of approximately 2 cm × 1 cm was tattooed by 30-times repeated two-second-lasting treatments with the tattoo needle oscillating at the voltage 17.4 V corresponding with the frequency 145 Hz (145 punctures per second) set on the power supply (DC POWER SUPPLY, DF 1730 SB3A, Bortech Tattoogrosshandel, Wuppertal, Germany). Thus, every tattooed mouse received during one immunization the total number of 60 900 (7 × 30 × 2 × 145 = 60 900) solid-needle punctures to deliver 50 μg DNA in 10 μl TE buffer or 121 800 (2 × 60 900 = 121 800) solid-needle punctures to deliver 100 μg DNA in 20 μl TE buffer. The tattoo procedure was well tolerated, however local trauma involving minor swelling and reddening of the skin was observed.

In addition, some mice were pretreated with 50 μl of cardiotoxin (10 μM, Latoxan, Valence, France) five days before the first DNA immunization in the loci of vaccination. Thus, cardiotoxin was applied either into the tibia anterior muscle by needle injection or to the dorsal skin by tattoo.

### ELISA

Blood of immunized mice was collected 10 days after the third and 9 days after the fourth DNA immunization. For detection and endpoint-titration assays of HPV 16 L1-specific antibodies an antigen capture ELISA was used. For this, microtiter plates were coated overnight at 4°C with 50 μl PBS containing purified rabbit polyclonal IgG anti-HPV16 L1 antibodies at a 1:200 dilution. Plates were blocked with 100 μl 3% milk/PBS-0.3% Tween 20 for 1 h at 37°C followed by the addition of 50 μl of the HPV16 L1 VLPs (5 mg/ml) diluted 1:1500 in 1.5% milk/PBS-0.3% Tween 20 for 1 h at 37°C. Plates were washed with PBS-0.3% Tween 20 and 50 μl of mouse serum were added in 2-fold dilutions starting at 1:50 and ending at 1:13107200 and incubated for 1 h at 37°C. Non-specific binding was determined using the dilution 1:50 of the mouse sera on plates coated with PBS only. Plates were washed and incubated with 50 μl/well of a sheep anti-mouse IgG polyclonal antibody conjugated to peroxidase (Sigma) diluted 1:3000 in 1.5% milk/PBS-0.3% Tween 20 for 1 h at 37°C. After the final washing, 100 μl/well of ABTS [2,2'-azino-bis(3-ethylbenz-thiazoline-6-sulfonic acid)] staining solution (1 mg/ml in a 100 mM sodium acetate-phosphate buffer, pH 4.2, 0.015% H_2_O_2_) was used for enzyme reaction. Absorptions were measured at 405 nm in a Titertek automated plate reader after 40–60 minutes.

### IFN-γ-enzyme-linked immunosorbent (ELISPOT) assay

The ELISPOT assay was performed 9 days after the fourth DNA immunization as described in our previous work [18]. MultiScreen IP sterile plates (96 well; Millipore, Eschborn, Germany) were pre-soaked with 70% ethanol for 1 min, and the ethanol was removed by extensive rinsing with PBS. The plates were coated with 600 ng per well of anti-mouse interferon gamma (IFN-γ) capture antibody (BD Pharmingen, Heidelberg, Germany) in 100 μl of PBS overnight at 4°C. Unbound antibody was removed by washing twice with PBS and twice with medium (RPMI-1640, Sigma; 10% fetal calf serum, 2 mM L-glutamine, 1% penicillin-streptomycin). Plates were blocked for 7 h with 100 μl of medium at 37°C, and splenocytes from individual mice were seeded in four serial dilutions: 2, 1, 0.5 and 0.25 × 10^6 ^cells per well in 100 μl of medium. Splenocytes from each mouse were left either untreated (background control), or stimulated with 900 ng of pokeweed mitogen (Sigma) in 100 μl of medium (positive control), or with 0.2 μM L1 aa165-173 peptide [19] in 100 μl of medium. Plates were incubated for 20 h at 37°C. Cells were removed by six washes with PBS-0.01% Tween 20 and one wash with sterile water. Then, 200 ng of sterile-filtered biotinylated rat anti-mouse IFN-γ detection antibody (BD Pharmingen) in 100 μl of PBS were added per well, and the plates were kept at 4°C overnight. The plates were washed six times with PBS-0.01% Tween 20 and once with PBS, and this was followed by the addition of 100 μl of a 1:1000 dilution of streptavidin-alkaline phosphatase (BD Pharmingen) in PBS. Plates were incubated for 30 min at room temperature and then washed three times with PBS-0.01% Tween 20, followed by three washing steps with PBS alone. Plates were developed with 5-bromo-4-chloro-3-indolylphosphate (BCIP/Nitro Blue Tetrazolium Liquid Substrate System; Sigma), 100 μl per well. The reaction was stopped after 15 minutes by rinsing the plates with water. Spots were quantified using an ELISPOT reader (AID EliSpot Reader ELR04; AID GmbH, Strassberg, Germany).

### Statistical analysis

Data of end-point titration of ELISA assay were analyzed by Wilcoxon Rank sum test. For ELISPOT assay analysis, we performed two tailed unpaired t-test using Prism 4 software (GraphPad Software, Inc., San Diego, CA, USA). A difference between groups was considered significant for p < 0.05.

## Results

**Figure 1:**
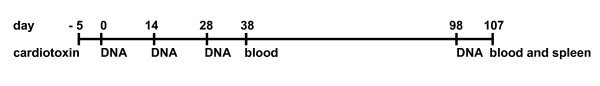
**Immunization scheme**. Mice were immunized four times with DNA on days 0, 14, 28 and 98. Cardiotoxin pre-treatment was carried out 5 days prior the first DNA immunization. Blood was collected twice, on days 38 and 107. Splenocytes were isolated on day 107 and analyzed by ELISPOT assay.

To compare different routes of delivery of DNA vaccines, i.e. intradermal tattooing versus intramuscular needle-injection, as well as the adjuvant effect of GM-CSF DNA co-delivery or cardiotoxin pre-treatment, we immunized mice with HPV16 L1 DNA four times as described in Material and Methods. The time-schedule of immunizations is outlined in Figure 1.

### DNA-tattooing induces higher levels of specific antibodies than DNA-intramuscular injection

**Figure 2:**
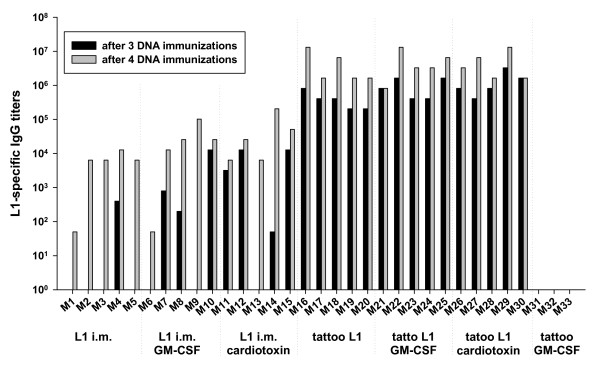
**VLP-based ELISA for detection of serum IgG antibody titers after DNA plasmid immunization**. Six groups of mice (5 per group) were immunized with HPV16 L1 DNA on days 0, 14, 28 and 98 either by tattoo or intramuscularly without any adjuvant, in combination with prior application of cardiotoxin or in mixture with mouse GM-CSF DNA (ratio 1:1). For control, a group of mice was tattooed with mouse GM-CSF DNA. The blood was collected after 3 and 4 immunizations for the estimation of L1-specific antibodies. The end-point titration of sera was performed. The titers of L1-antibodies were determined using an absorption value of 0.4 as cut-off for ELISA.

**Figure 3:**
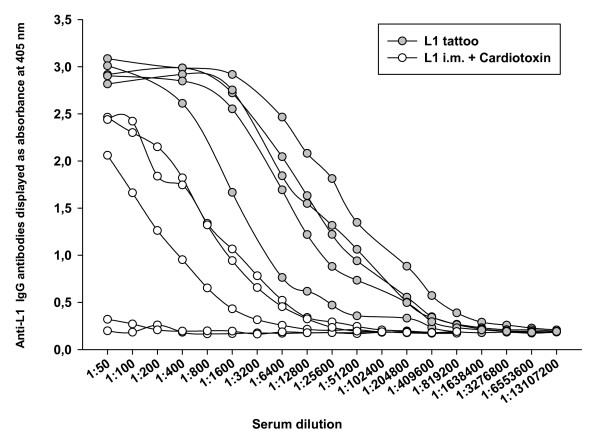
**End-point titration of sera**. To show the values of end-point titration of sera from individual mice, we chose two groups of mice immunized three times with HPV16 L1 DNA either intramuscularly after cardiotoxin pre-treatment or by tattoo. Mice immunized intramuscularly with HPV 16 L1 DNA after cardiotoxin pretreatment developed lower levels of L1-specific antibodies than L1-tattooed mice. Serum values below the ELISA cut-off value of 0.4 optical density (O.D.) at 405 nm were considered to be negative.

After three immunizations, all mice (15/15) immunized by HPV16 L1 DNA-tattooing developed high levels of L1-specific antibodies, while intramuscular delivery of DNA induced L1-specific antibodies only in 8 out of 15 mice: in one mouse receiving no adjuvant (1/5), three mice co-immunized with GM-CSF DNA (3/5) and four mice pre-treated with cardiotoxin (4/5; Figure 2). The end-point titration of sera collected after three immunizations showed that the level of L1-specific antibodies was 500–2000 times higher in all five mice immunized three times by tattoo (without adjuvant) than the titer of the single antibody-positive mouse of the group immunized intramuscularly without adjuvant (Figure 2). Moreover, three doses of DNA delivered by tattoo induced at least 16-times higher levels of anti-L1 antibodies than three intramuscular DNA immunizations applied after cardiotoxin pre-treatment or using GM-CSF DNA co-delivery (Figures 2 and 3). Comparing groups of mice immunized with DNA using the two different delivery methods, all of the tattooed mice produced significantly higher levels of specific antibodies than intramuscularly immunized mice after three immunizations (p < 0.0001).

The fourth DNA immunization increased the number of mice producing L1-specific antibodies in the intramuscularly immunized group (from 8/15 to 15/15 positive mice) and also enhanced the level of L1-specific antibody production in 14 out of the 15 mice treated with the tattoo device. The boosting effect of the fourth DNA immunization was higher in intramuscularly-immunized than in tattooed mice. However, four intramuscular DNA immunizations induced still lower production of L1-specific antibodies than three DNA immunizations delivered by tattoo (p < 0.0001).

Both GM-CSF DNA co-delivery and cardiotoxin pre-treatment enhanced the L1-specific humoral responses after both three and four HPV16-L1 DNA immunizations delivered either by intramuscular injection or tattoo, but the differences were not statistically significant. The effect of both adjuvants (GM-CSF DNA co-delivery and cardiotoxin pre-treatment) was more pronounced in mice immunized intramuscularly than tattooed and in mice immunized three times rather than four times.

No specific anti-L1 antibodies were detected at any dilution in sera of the control group of mice receiving GM-CSF DNA delivered by tattoo.

### DNA-tattooing induces higher specific cellular immune responses than DNA-intramuscular injection

**Figure 4:**
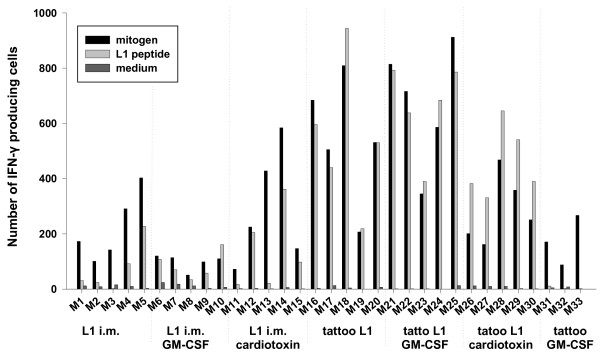
**Cytotoxic T-cell response in DNA immunized mice detected by IFN-γ-ELISPOT assay**. Cellular immune responses after four DNA vaccinations are shown. Six groups of mice (5 per group) were immunized with HPV16 L1 DNA on days 0, 14, 28 and 98 either by tattoo or intramuscular delivery without any adjuvant, in combination with prior application of cardiotoxin 5 days before the first immunization or in mixture with mouse GM-CSF DNA (1:1). A control group of three mice was tattooed with mouse GM-CSF DNA. Splenocytes were isolated 9 days after the last DNA immunization and examined in 4 serial dilutions in the IFN-γ-ELISPOT assay. The representative numbers of spots reflecting IFN-γ-producing cells per 250,000 splenocytes are shown. Splenocytes were stimulated non-specifically with mitogen or specifically with the L1 peptide (aa 165–173). Non-stimulated splenocytes were used as negative controls.

Nine days after the fourth immunization, the splenocytes from all vaccinated mice were analyzed by an L1-specific IFN-γ-ELISPOT assay. The non-specific stimulation with mitogen led to the enhancement of IFN-γ-producing cells in all mice, showing that the splenocytes used in the ELISPOT assay were alive and able to secret IFN-γ (Figure 4). The numbers of cells producing IFN-γ per 250 000 splenocytes after mitogen-stimulation ranged from about 90 to 270 in the control group of three mice (GM-CSF-tattooed mice), about 50 to 600 for the L1-intramuscularly immunized mice (difference is non-significant) and about 200 to 900 for the L1-tattooed mice (p < 0.05). The non-specific, mitogen-induced increase of IFN-γ-producing cells in splenocytes of the L1-tattooed mice was significantly higher in comparison with the L1-intramuscularly immunized mice (p < 0.001).

The comparison of the numbers of IFN-γ-producing cells in serial dilutions of splenocytes incubated one day with either plain medium or in the presence of an L1 peptide (aa165–173; [19]) revealed that one mouse (M3) immunized intramuscularly with HPV16 L1 DNA and all three control mice immunized with GM-CSF DNA did not elicit detectable L1-specific cellular responses. The numbers of L1-specific IFN-γ-producing cells per 250 000 splenocytes ranged from 3 to 362 for the 15 mice that received the HPV16 L1 DNA by intramuscular injection (not statistically different in comparison with the control mice tattooed with GM-CSF DNA) and ranged from 219 to 944 cells for the L1-tattooed mice (P < 0.001, Figure 4). The L1-specific cellular immune responses detected in the L1-tattooed mice were significantly higher than that in the mice immunized with L1 intramuscularly (P < 0.0001).

The effects of the cardiotoxin pre-treatment or the GM-CSF co-delivery on L1-specific cellular immune responses elicited after HPV16 L1 vaccination were not significant. Both adjuvants enhanced the numbers of L1-specific IFN-γ-producing cells in mice immunized with L1 or GM-CSF intramuscularly as well as in the L1-tattooed mice (not-significant). The L1-tattooed mice that were pre-treated with cardiotoxin showed lower numbers of both mitogen- and L1-peptide-stimulated IFN-γ-producing splenocytes than the L1-tattooed mice receiving no prior treatment with cardiotoxin (not statistically significant).

## Discussion

In this study we compared different protocols of DNA immunization and observed that three DNA immunizations delivered by tattoo elicited much higher specific humoral immune responses than three or even four intramuscular injections. Further, tattooing induced higher specific cellular immune responses than intramuscular DNA injections. Administration of an adjuvant (GM-CSF or cardiotoxin) had virtually no effect on the efficacy of tattoo immunization whereas it enhanced the effect of the intramuscular injection.

The cardiotoxin pre-treatment of muscles before administration of DNA is a routinely performed procedure for DNA immunization. In this work, we evaluated the importance of cardiotoxin pre-treatment for induction of anti-L1 specific antibodies. It has been shown that some intramuscularly delivered DNA vaccines are not able to induce effectively specific antibody responses without the support of cardiotoxin [20], while for other DNA vaccines the usefulness of muscle pretreatment was not demonstrated [21]. We immunized mice three times with 50 μg pUF3-hL1 DNA in 2-week intervals and found that mice more consistently developed L1-specific antibodies after cardiotoxin administration than receiving no muscle pre-treatment (4/5 versus 1/5). Further, four intramuscular immunizations with 50 μg pUF3-hL1 DNA elicited L1-specific antibodies in all mice regardless of the use of cardiotoxin, indicating that the absence of cardiotoxin pre-treatment of muscles might be substituted by increasing the number of boosting DNA immunizations.

To our knowledge, there are only four studies addressing the use of tattooing for DNA immunization [6-8,22] and only one of the publications focuses on a comparison of tattooing with intramuscular needle injection of DNA [6]. In this work, we observed that the tattoo delivery induced more robust immune responses than intramuscular delivery that was in concordance with previous findings of Bins and coworkers [6]. However, in our study we used higher doses of DNA for tattoo delivery and also a more intensive tattoo protocol than Bins et al., suggesting that reducing the dose of DNA and mild conditions of tattooing could result in a decrease of efficiency of DNA tattoo immunization. Although we did not determine the mechanisms by which DNA tattooing leads to better immune response one can speculate that this is due to (i) better uptake of the DNA by non-antigen-presenting cells [22], (ii) better uptake of DNA by antigen-presenting cells, (iii) duration of expression or (iv) the induced traumata accompanying the tattooing [3]. The fact that the lymphocytes from mice treated with the tattoo device demonstrated a higher mitotic index when treated with a mitogen supports the idea of induction of traumata and release of danger signals. We observed that treatment of mice with the tattoo device induced local trauma which was evident macroscopically by minor swelling and reddening of the punctured skin areas and was also reflected in stronger T-cell responses towards an unspecific mitogen, detected in the ELISPOT assay. Interestingly, this effect was only observed in animals that had received the L1 construct but not or to a much lower extent in the control mice treated with the GM-CSF expression vector alone. Perhaps, the viral origin of the L1 protein and/or the high immunogenicity of L1-virus-like particles contributed to non-specific stimulation of murine immune system.

The mode of DNA delivery (tattooing versus intramuscular injection) had a much higher effect on the vaccination efficiency than the addition of adjuvants (GM-CSF, cardiotoxin). Similarly, another DNA delivery method, intramuscular *in vivo *electroporation, has been shown to induce higher antibody titers than intramuscular DNA injection in combination with cardiotoxin pretreatment [20]. It is conceivable that a robust local tissue injury induced by tattooing attracts leukocytes and leads to local release of cytokines [3]. The exact mechanisms of action of cardiotoxin are not yet determined but tissue damage and necroses are important factors [23]. The GM-CSF attracts antigen-presenting cells to the application site [24]. Thus, tattooing may partially substitute for the function of cardiotoxin and GM-CSF in their function. This is consistent with the observation that cardiotoxin pre-treatment or co-administration of the GM-CSF expression construct did not have any effect on tattoo immunization. The intramuscular needle-injection causes very little tissue damage [25]. That could be the reason why both GM-CSF and cardiotoxin substantially enhanced the immune responses after intramuscular DNA immunization.

The advantage of tattoo treatment is the low price of the tattoo device and a standardized method for the application; the main disadvantages are the strain on the animals and a somewhat cumbersome application procedure. In particular, the local traumata induced by the tattooing procedure might not be considered acceptable in routine prophylactic vaccination settings involving human subjects. Nevertheless, DNA vaccination via tattoo seems to be the method of choice if faster and stronger immune responses have to be achieved. Potential applications might be vaccination of life stock for prophylaxis or of human beings for therapeutic purposes.

## Competing interests

The author(s) declare that they have no competing interests.

## Authors' contributions

DP and IR performed the experiments. DP and MM wrote the paper. MM designed the study. All authors read and approved the final manuscript.
